# Effect of Escin Alone or in Combination with Antifungal Agents on Resistant *Candida glabrata* Biofilms: Mechanisms of Action

**DOI:** 10.3390/antibiotics12071210

**Published:** 2023-07-20

**Authors:** Angela Maione, Marianna Imparato, Marilena Galdiero, Elisabetta de Alteriis, Antonia Feola, Emilia Galdiero, Marco Guida

**Affiliations:** 1 Department of Biology, University of Naples ‘Federico II’, Via Cinthia, 80126 Naples, Italy; angela.maione@unina.it (A.M.); dealteri@unina.it (E.d.A.); antonia.feola@unina.it (A.F.); 2 Department of Experimental Medicine, University of Campania “Luigi Vanvitelli”, 81100 Naples, Italy; marilena.galdiero@unicampania.it; 3 National Biodiversity Future Center (NBFC), 90133 Palermo, Italy; 4 Center for Studies on Bioinspired Agro-Environmental Technology (BAT Center), 80055 Portici, Italy

**Keywords:** biofilm, *Candida glabrata*, azole resistance, β-escin, antifungal therapy

## Abstract

Nowadays, the increase in antimicrobial-resistant fungi (AMR) is certainly a major health concern, and the development of alternative therapeutic strategies has become crucial. Natural products have been used to treat various infections, and their chemical properties contribute to the performance of their biological activities, such as antifungal action. The various virulence factors and mechanisms of resistance to antifungals contribute to making *Candida glabrata* one of the most frequent agents of candidiasis. Here we investigate the in vitro and in vivo activity of β-escin against *Candida glabrata*. The β-escin MICs were determined for a reference strain and two clinical isolates of *C. glabrata*. Furthermore, growth kinetics assays and biofilm inhibition/eradication assays (crystal violet) were performed. The differences in the expression of some anti-biofilm-associated genes were analyzed during biofilm inhibition treatment so that reactive oxygen species could be detected. The efficacy of β-escin was evaluated in combination with fluconazole, ketoconazole, and itraconazole. In addition, a *Galleria mellonella* infection model was used for in vivo treatment assays. Results have shown that β-escin had no toxicity in vitro or in vivo and was able to inhibit or destroy biofilm formation by downregulating some important genes, inducing ROS activity and affecting the membrane integrity of *C. glabrata* cells. Furthermore, our study suggests that the combination with azoles can have synergistic effects against *C. glabrata* biofilm. In summary, the discovery of new antifungal drugs against these resistant fungi is crucial and could potentially lead to the development of future treatment strategies.

## 1. Introduction

In the last decades, resistance of most human pathogenic fungi to conventional drugs has rapidly increased, representing a severe challenge to global public health and leading to an increase in mortality rates, hospitalization time, and health care costs [1,2].

Drugs for the treatment of fungal infections are limited, with only four classes of antifungal compounds targeting different structures with efficacy damaged by toxicity or drug resistance [3].

*Candida glabrata* is a human commensal yeast able to colonize mucosal surfaces such as the oral cavity and the gastrointestinal tract of healthy people. This yeast, under certain predisposing conditions, behaves as an opportunistic pathogen, causing both superficial and systemic infections. Indeed, *C. glabrata* is the second most frequent cause (15–20% of cases) of invasive candidiasis in most European countries [4], and it is notable for causing recalcitrant infections. The incidence of such infections is now increasing, showing overall greater resistance to common antifungal agents, especially azoles [5].

*C. glabrata*, despite the lack of morphological flexibility, is often associated with biofilm formation due to its attachment to host cells, which is partly mediated by a large family of adhesins. Biofilm occurrence is one of the main virulence factors associated with increased resistance to antifungal agents [6,7,8].

Biofilms represent the chosen lifestyle of microorganisms and are present in more than 80% of all human infections [9,10]. Biofilm-embedded microorganisms show up to 1000-fold higher resistance to antimicrobials compared to their planktonic counterparts [11], thanks to the protective biofilm matrix properties and metabolic dormancy [12]. Microorganisms in biofilms remain robust, leading to treatment failure and recurring infections. Therefore, discovering new and effective alternative substances with potential antimicrobial and anti-biofilm activities is fundamental because they could effectively combat not only planktonic but also sessile fungal cells [13].

Currently, the main methods for overcoming fungal resistance involve not only the development of novel antifungal drugs but also combining existing antifungal drugs with non-antifungal agents, which is one of the solutions to overcome the current challenges [14].

The decrease in therapeutic options for the treatment of infections has led to the need to study the antimicrobial action of plant products. The inhibitory activities of a considerable number of plant-derived compounds against many fungal pathogens have been widely reported when used alone or in combination with traditional drugs [15].

Saponins from different plant species are one of the most common and diffused groups of phytoanticipins involved in plant defense via their antimicrobial potency. They exhibit relevant antifungal activity against both yeasts and filamentous fungi [16,17,18].

β-escin is one of the principal constituents of the natural mixture of triterpene saponins (called escin) obtained from *Aesculus hippocastanum* L. (*Hippocastanaceae*) seed extracts. β-escin is largely used for its beneficial role in clinical therapy due to its anti-edematous, anti-inflammatory, and antioxidative effects [19]. It has also been demonstrated as having virucidal and broad spectrum anti-viral activities against the enveloped viruses HSV-1, VSV, and Dengue [20,21,22], as well as an antifungal effect against yeasts, as shown by the disk diffusion method [23]. 

The objective of this study was to assess the antifungal effect of β-escin not only against planktonic *Candida* cells but also as an anti-biofilm agent by investigating its effect on the formation and destruction of mature biofilms in two antifungal-resistant clinical isolates of *C. glabrata.* Changes in biofilm formation were investigated to elucidate the anti-resistance mechanisms of β-escin. Therefore, during biofilm inhibition, the β-escin effect was evaluated on the expression of some key genes (*ERG11, ALS3, CDR1, HOG1, and FKS1*) involved in *C. glabrata* virulence, as well as on the production of ROS. 

Escin interacts with the cell membranes of mammalian cells, as shown by electrophysiological studies on CHO cells, and leads to the formation of transmembrane pores, permitting the permeation of large molecules such as nucleotides and drugs [24]. In the case of *C. glabrata*, we assessed the effect of β-escin on cell membrane integrity by fluorescence-activated cell sorting (FACS) analysis, evaluating yeast cell permeability to propidium iodide following β-escin exposure.

Starting from this observation, we exploited the permeabilization/perforating properties of β-escin to potentiate the effect of conventional antifungals against *C*. *glabrata*. Indeed, different drugs or compounds in combination with antifungal are recommended to inhibit *C. Glabrata*-resistant strains [25], but only in one study [18] was the β-escin used in combination with the conventional drug nystatin against some clinical isolates of *C*. *glabrata*. In this work, we checked the combination of β-escin with three azoles commonly used for the treatment of fungal infections, assessing a possible synergistic effect in vitro.

Further, using a *Galleria mellonella* model, the combined effects of β-escin with azole drugs were analyzed in order to confirm their efficacy also in vivo.

## 2. Results

2.1. β-Escin Cytotoxicity Evaluation

The effect of β-escin on cell viability was detected via the colorimetric assay 3-(4,5-dimethylthiazol-2-yl)-2,5-diphenyl-2H-tetrazolium bromide (MTT). Human keratinocytes (HaCaT) were incubated with different concentrations of the compound (ranging from 0.5 to 20 μg mL^-1^).

**Figure 1:**
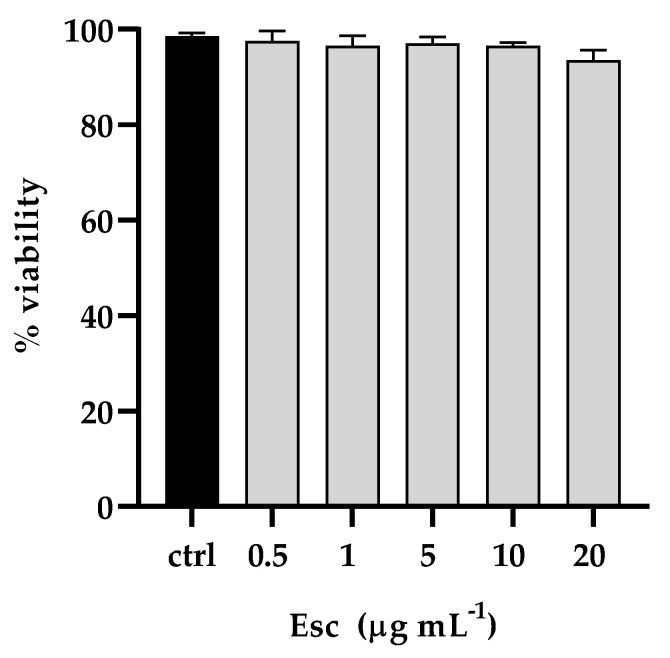
Determination of cytotoxic activity of β-escin shown as the percent cell viability of the HaCaT cell line treated with different concentrations for 24 h. The assays were performed in three independent experiments. Standard deviations are always less than 10%.

The cytotoxic activity was assessed by monitoring cell viability and expressed as a percentage of viability relative to the viability of untreated cells. As reported in Figure 1, the MTT assay revealed that the survival of treated cells was comparable to that of control untreated cells, showing that β-escin did not present significant cytotoxicity to the tested cell lines under these experimental conditions.

### 2.2. Disk Diffusion Test

The disc diffusion assay was performed to get the antimycotic susceptibility profiles of *C. glabrata* DSM 1226 and the two *C. glabrata* clinical isolates and are reported in Table 1. *C. glabrata* DSM1226 strains tested in this study were susceptible to FLC, KET, and ITZ, while the clinical isolate C18 was multi-resistant to all the tested drugs. The clinical isolate C27 exhibited, on the contrary, intermediate susceptibility to FLC and ITZ and resistance to KET.

### 2.3. Antifungal Activity (MIC-MFC)

The antimicrobial activity of β-escin was tested against *C. glabrata* DSM 1226 and the two clinical isolates. The compound resulted in inhibiting all the tested *C. glabrata* strains: the MIC value was 80 μg mL^−1^ for the reference strain and 40 μg mL^−1^ and 50 μg mL^−1^, respectively, for the clinical isolates C18 and C27 (Table 2). The MFC values were analyzed to understand if the β-escin exhibited a fungicidal action. The MFC corresponds to the lowest concentration capable of causing the elimination of more than 99.9% of microorganisms. Results are reported in Table 2 and showed that β-escin exerted fungicidal activity against all three strains tested because the ratio MFC/MIC was lower than 4.

### 2.4. Time–Growth Curves and Time–Kill Curves 

**Figure 2:**
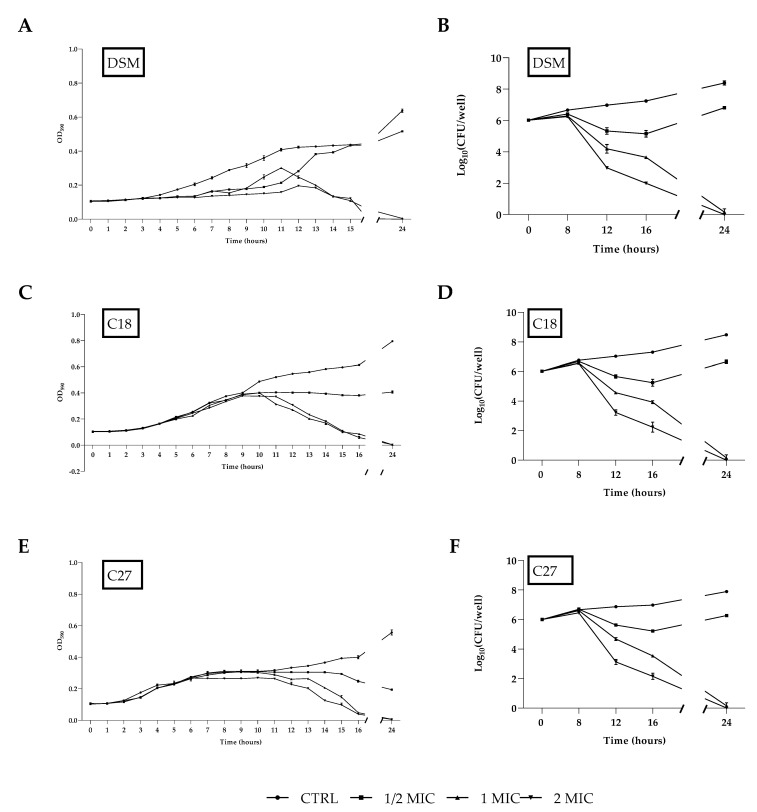
Growth curves of *C. glabrata* DSM 1226, C18, and C27 strains (**A**,**C**,**E**) treated with β-escin at concentrations 1/2 × MIC and 1 × MIC and 2 × MIC compared to non-treated cells. Time to kill curves (**B**,**D**,**F**) of the same strains treated with β-escin at the same concentrations and compared to non-treated. Data are reported as mean of three independent experiments ± SD.

*C. glabrata* DSM 1226, C18, and C27 strains were treated with different concentrations of β-escin to construct time-growth curves. As shown in Figure 2A,C,E, β-escin concentrations corresponding to 1/2 × MIC, 1 × MIC, or 2 × MIC significantly decreased yeast growth compared to the control culture. The time-killing assay highlighted the fungicidal effect of β-escin at 1 × MIC and 2 × MIC (Figure 2B,D,F) since a significant decrease in the viable cell number was observed with a reduction of about 2 log after 16 h of incubation with β-escin at 1 × MIC concentration, and a complete microbicide effect was observed after 24 h.

### 2.5. Biofilm Activity

**Figure 3:**
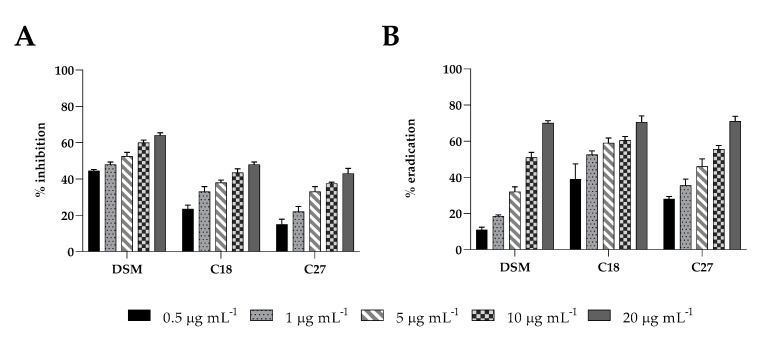
The effect of β-escin on the biofilms of *C. glabrata* DSM11226 and *C. glabrata* clinical isolates C18 and C27. Inhibition (panel (**A**)) and eradication (panel (**B**)) quantified with crystal violet after 24 h of treatment with different doses of β-escin.

Insensitivity to antifungals is partially due to biofilm formation, which limits drug penetration and makes cells more resistant to antimicrobial drugs. Here, we examined the inhibitory effect against biofilm formation and the eradication capability on pre-formed biofilms of β-escin at sub-MIC concentrations using the CV assay (Figure 3A,B). β-escin inhibited the establishment of *C. glabrata* DSM1226 biofilm in a dose-dependent manner, reaching at the highest concentrations tested (20 μg mL^−1^) an inhibitory effect of approximately 65% (*p* < 0.05). A lower impact on C18 and C27 strains was observed. β-escin at a concentration of 20 μg mL^−1^ led to a 45% and 40% inhibition ability, respectively. All the *C. glabrata* strains showed reduced mature biofilm in the presence of at the same sub-MIC concentrations of β-escin, with a maximum of 75% eradication when 20 μg mL^−1^ of β-escin were used.

### 2.6. β-Escin Differentially Affects Gene Expression in Fungal Biofilm 

**Figure 4:**
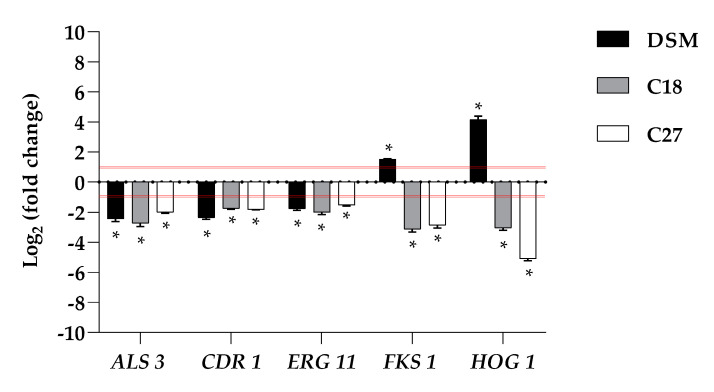
Expression analysis of selected genes (*ALS3*, *CDR1, ERG11*, *FKS1,* and *HOG1*) of *C. glabrata* DSM 1226, C18, and C27 strains using real-time qPCR in response to β-escin. Histograms represent the fold differences in the expression levels of the genes selected during inhibition of biofilm with β-escin at concentration of 5 µg mL^-1^. Red lines show fold change thresholds of -1 and +1, respectively. * *p* < 0.05 indicate fold changes significantly different from untreated samples (*t*-test).

Because it is important to understand whether the inhibitory effect of β-escin on biofilm formation is related to changes in some biofilm-related genes, transcriptional analysis of the ergosterol synthesis gene (*ERG11*), 1,3-β-glucan synthase (*FKS1*), adhesin (*ALS3*), multidrug transporter (*CDR1*), and kinase involved in the response to osmotic stress (*HOG1*) genes was investigated by qRT-PCR upon treatment of β-escin at sub-lethal concentration, as summarized in Figure 4.

The data obtained revealed that the expression of these selected *C. glabrata* genes was differentially affected. Our study revealed that the expression levels of *ALS3* showed differential behavior upon treatment with β-escin, they were moderately downregulated in C27 and significantly downregulated in C18 and in the reference strain. β-escin downregulated the *ERG11* gene in all three strains studied. Likewise, *CDR1* was also found to be significantly downregulated in DSM 1226 and C18. Contrariwise, *FKS1* was downregulated in the two clinical strains and not detected in the reference strain. In the case of *HOG 1*, which mainly regulates some stress response pathways, it was downregulated in the two clinical isolates while upregulated in DSM 1226.

### 2.7. Treatment-Induced Oxidative-Damage in C. glabrata Biofilm

**Figure 5:**
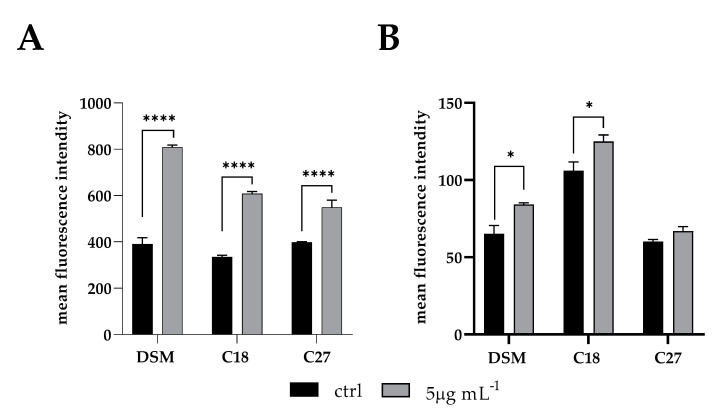
Intracellular ROS overproduction detected using DCFH-DA (panel (**A**)) and mitochondrial ROS detected using MitoSOX Red (panel (**B**)) during inhibition of *C. glabrata* biofilms of DSM 1226, C18, and C27 strains by 5 mg mL^−1^ β-escin. * *p* < 0.05, **** *p* < 0.0001 (Tukey’s test).

Once we assessed that β-escin exerted a fungistatic/fungicidal effect on *C. glabrata* cells, we presumed that ROS-induced oxidative stress may be part of such activity. The fluorescent dye 2,7-dichlorodi hydrofluorescein diacetate (DCFH-DA) and the MitoSOX Red were used to detect the intracellular ROS and mitochondrial-specific ROS levels, respectively, during biofilm inhibition of *C*. *glabrata* by sub-MIC β-escin concentration (5 μg mL^−1^). Increased levels of both intracellular and mitochondrial ROS were recorded in all three *Candida* strains when exposed to β-escin (Figure 5).

### 2.8. FACScan Analysis 

**Figure 6:**
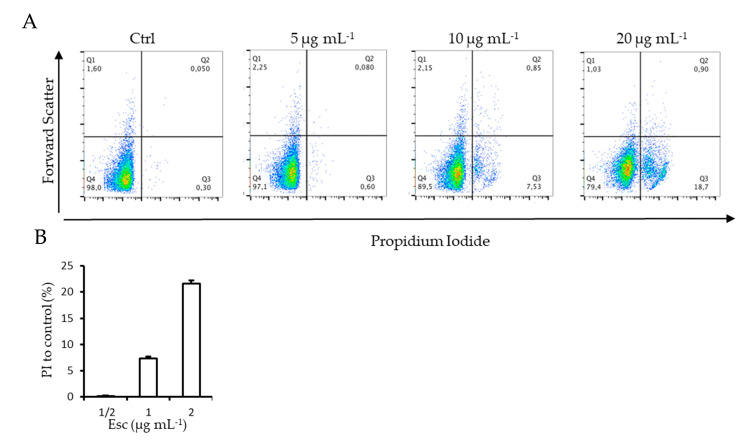
FACS analysis revealed an increase in cell membrane permeability induced by β-escin. *C. glabrata* cells were treated with β-escin at the concentrations of 5 µg mL^-1^, 10 µg mL^-1^, and 20 µg mL^-1^ for 18 h. Fungal cells without β-escin were used as negative control. Dot plot images (**A**) were analyzed by FlowJo 8.7 software. The histograms (**B**) represent the % of cell positive to PI staining compared to control.

Cell membrane integrity of *C. glabrata* cells during inhibition of biofilm formation in the presence of β -escin was evaluated by using the DNA-intercalating dye propidium iodide (PI) by FACS Analysis. The increase in fluorescence intensity indicated that β-escin induced the incorporation of PI into the cells in a dose-dependent manner. As shown in Figure 6, the lowest dose (5 µg mL^−1^) did not alter cell membrane permeability compared to the control; instead, 10 µg mL^−1^ and 20 µg mL^−1^ doses determined an increase in cells positive for PI stain of 7.4 ± 0.3 and 22 ± 0.6%, respectively (Figure 6B). Moreover, treated cells seemed to be larger than the untreated control cells when seen in FACS parameter, demonstrating that the promotion of membrane permeability mediated by β-escin led to an increase in cell volume. 

### 2.9. Evaluation of Synergistic Effect of β-Escin 

**Figure 7:**
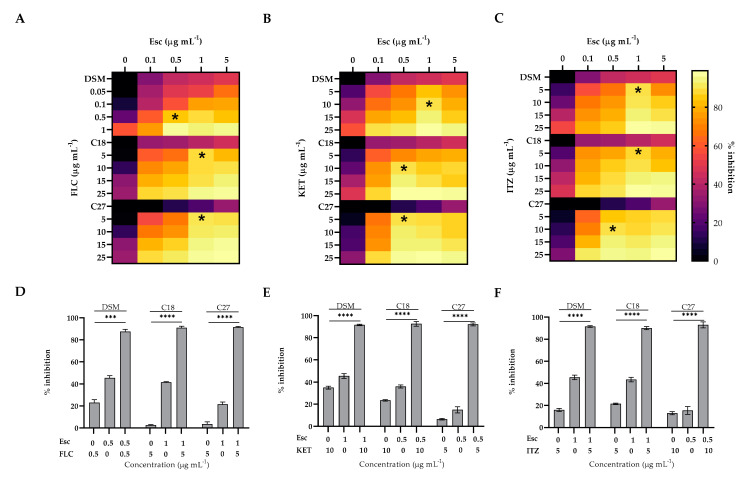
Synergistic effect of β-escin/azole combination against the biofilms of *C. glabrata* DSM 1226, C18, and C27 strains. Heat-maps show the synergistic effect of Esc/FLC (panel (**A**)), Esc/KET (panel (**B**)), Esc/ITZ (panel (**C**)); * indicate the synergistic effect (FICI ≤ 0.5). The corresponding percentage of biofilm inhibition for Esc/FLC (panel (**D**)), Esc/KET (panel (**E**)), and Esc/ITZ (panel (**F**)) are shown. Data are means of three independent experiments. *** *p* < 0.001, **** *p <* 0.0001 (Tukey’s test).

After evaluating the anti-biofilm efficacy of β-escin at different concentrations during the inhibition assay, the checkerboard assay was performed to analyze the possible synergistic activity of β-escin with FLC, ITZ, and KET. The synergistic effects for each strain, evaluated by the calculation of the FICI of each combination, are reported in Figure 7A–C with the corresponding values of biofilm inhibition as determined by the crystal violet assay (Figure 7D–F. The results showed a synergistic effect of the β-escin with all the three azoles tested, and for all the three strains examined, it reached an inhibition capability of 90% in comparison to mono-treatment by each azole. These results indicated that the inhibition effect of β-escin (Figure 3) could be further improved by combining it with azoles that reinforce their activity.

### 2.10. Survival Rate 

Recently, the fungal infection model represented by *G. mellonella* has been largely used in the assessment of fungal virulence and antifungal drug efficiency. 

**Figure 8:**
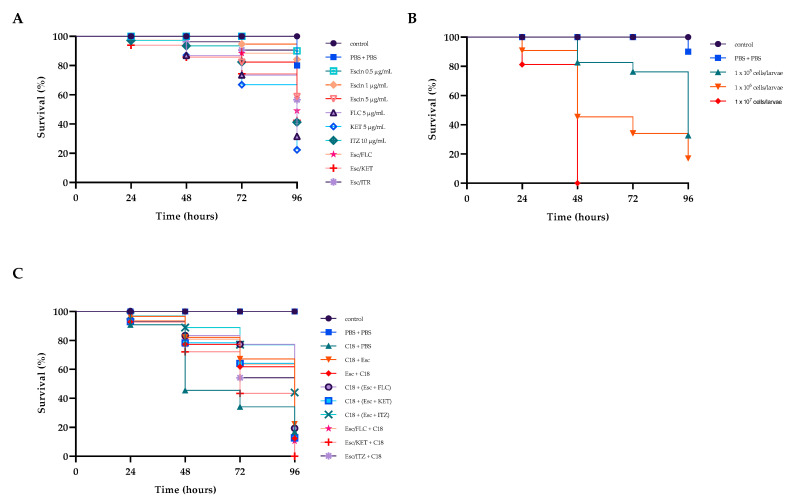
In vivo experiments in *Galleria mellonella*. Toxicity of β-escin and azole drugs alone or in combination (panel (**A**)). Determination of a suitable concentration of C18 per larva (panel (**B**)). Prevention/treatment of larvae inoculated with the three combinations Esc/FLC, Esc/KET, and Esc/ITZ (panel (**C**)). Control larvae were inoculated with 10 μL of PBS or intact. (N = 20 for each experiment and condition).

β-escin had no toxic effect on *G. mellonella* (Figure 8A), and this result positively correlated with those from in vitro studies on HaCat cell lines where no cytotoxicity was detected (Figure 1).

To assess the virulence of the C18 strain on larvae, their survival was calculated by injecting concentrations of 10^5^, 10^6^, or 10^7^ CFU of fungal cells per larva. Larval survival was monitored for four days after infection (Figure 8B). After 48 h, all larvae injected with 10^7^ CFU/larva died. After four days, survivals of about 80 and 40% were recorded for inoculum concentrations of 10^5^ and 10^6^ CFU/larva, respectively. Therefore, a concentration of 10^6^ CFU/larva was selected for the subsequent tests.

Then, we determined whether the drug combinations Esc/FLC, Esc/KET, and Esc/ITZ had an influence on the survival rates of larvae. We found that all the combinations led to extended survival compared to the drug alone or the control group. In detail, β-escin did not exert significantly toxic effects on the larvae when administered at concentrations of 5 μg mL^−1^ up to 48 h (Figure 8A) keeping a 60% survival rate at 96 h. Each of the three azole drugs showed about 20% survival after 4 days. Instead, the three combinations improved larval survival rates by 20%, 20%, and 15%, respectively, compared to the mono-treatment of each drug, indicating the potential in vivo efficacy of this drug combination on resistant C18. Finally, we tested the protective/therapeutic effects of β-escin on C18 infected larvae. As shown in Figure 8C, after infection, the treatment with each combination prolonged the survival time of *G. mellonella* (Figure 8C). The survival rate of *G. mellonella* was significantly improved to about 30% with the treatment of Esc/ITR combination, indicating a strong antifungal activity of this combination in vivo.

## 3. Discussion

Over the past few years, there has been a significant increase in the frequency of fungal infections in humans, especially in immunocompromised cancer patients.

Even if *C. albicans* is still the most common cause of invasive candidiasis, the incidence of *C. glabrata* infections is growing, as it is now the second most common causative pathogen of infection [4]. In 2022, the World Health Organization (WHO) published a list of fungal “priority pathogens,” strengthening the global response to fungal infections and antifungal resistance. *C. glabrata* has been the first *Candida* species considered important to public health. *C. glabrata* strains easily and quickly acquire drug resistance to azoles, and some strains have an intrinsic resistance to azoles too [26]. FLC, ITZ, and KET are common azole drugs used for the treatment of *Candida* infections due to their high bioavailability. Unfortunately, their extensive and frequent use has gradually increased the number of drug-resistant strains, which represents a great challenge in the treatment of fungal infections.

Single-drug and multi-drug resistance in *C. glabrata* is becoming increasingly prevalent and difficult to treat. The current antifungal arsenal remains inadequate. Recent studies have reported that combining different drugs with azole therapy can result in strong synergistic effects, so the discovery of novel synergistic combinations could give a panel of alternatives to realize stability, low toxicity, low cost, and resistance advantages in antifungal applications [27,28].

A potential alternative to conventional antifungals in the treatment or prevention of candidiasis could be the study of natural resources, which have a different mechanism of action, a low incidence of side effects, and a different activity. In this study, a potential pharmacological strategy in anti-biofilm therapy was investigated, specifically the combination of conventional antifungals with β-escin. Escin is the active component of *Aesculus hippocastanum* used in traditional medicine to treat certain conditions, including hemorrhoids, varicose veins, hematomas, and venous congestion with anti-edematous, anti-inflammatory, and venotonic properties [29]. The total extracts of *Aesculus hippocastanum* have been shown to possess not only anti-bacterial activity towards *Staphylococcus aureus, Escherichia coli*, and *Streptococcus mutants* [22] but also antimycotic activity [21]. Despite the fact that β-escin in combination with nystatin showed an antifungal effect on *C. glabrata* [18], to date, there has been no research on the combined anti-biofilm effects of β-escin with other antifungal drugs.

In this study, we report the in vitro effect of β-escin against both planktonic growth and biofilm formation and the eradication of two azole-resistant isolates of *C. glabrata*. Furthermore, the effects of this antifungal agent were assessed in an in vivo invertebrate treatment model, also evaluating a possible synergistic mechanism of azoles (FLC, KET, and ITZ) combined with β-escin. In our in vitro experiments, we reported the β-escin MICs of the reference strain and the two clinical isolates C18 and C27 resistant to the drugs tested showing a fungicidal effect exerted after 24 h confirmed by the time–growth and time–kill curve assays. The pathogenesis of *C. glabrata* infection is mediated by a variety of virulence factors, including the adherence capacity, leading to biofilm formation on biotic and abiotic surfaces [4]. Here, we found that β-escin inhibited biofilm formation and destroyed pre-formed biofilms in all three strains tested in a dose-dependent manner, reaching at least 60% and 70% values, respectively, at the highest doses. Membrane integrity damage modulates other membrane functions, such as transport and signal transduction, and also induces oxidative stress [30]. In this work, we demonstrated by FACS analysis that β-escin affected membrane integrity of *C. glabrata* cells in a dose dependent manner indicating that β-aescin 1 × MIC and 2 × MIC increased in about 7.4% and 22% of cells, respectively, compared to the control. In accordance with this result, we also found that *Candida* cells exposed to β-escin during biofilm inhibition exhibited a higher level of ROS. As is known, an overproduction of ROS can lead to oxidative stress with the accumulation of intracellular hydroxyl radicals, which is the key factor in cell apoptosis [31]. The increased detection of iROS and mROS during biofilm inhibition confirmed their involvement when β-escin was used on resistant *C. glabrata*, suggesting that these mechanisms utilized by different antifungal agents is involved here as well. 

The qRT-PCR analysis further confirms the anticandidal activity of β-escin. The downregulation of *ERG11* and *ALS3* during biofilm inhibition suggested that this compound had potential for further development as part of novel anti-biofilm strategies. The down-expression of the efflux pump gene *CDR1* in all three fungi confirmed the impossibility of reducing the accumulation of intracellular β-escin during biofilm inhibition. As reported, the *HOG* pathway is activated in response to hyperosmotic stress, leading to a rapid and transient activation of expression of many genes. β-escin treatment induced *HOG1* activation in the reference strain, showing an increase in fungal susceptibility to this antifungal compound, but unexpectedly not in C18 and C27. Similar results have been reported for other fungal pathogens treated with different antifungal reagents.

Recently, drug combinations have become an effective strategy to overcome the increasing resistance of fungal infections. In this work, we exploited the assessed potentiality of β-escin to affect membrane integrity and consequent permeability of *C. glabrata* cells and combined it with conventional azoles to favor the inhibition of biofilm formation. Our results clearly indicated that, in combination with azoles, β-escin had a positive impact on both *C. glabrata* reference strains and resistant isolates. The three different combinations exhibited more effective activity during biofilm inhibition than the mono-treatment, indicating β-escine/azole as a potential therapeutic combination against resistant *C. glabrata* biofilm infections.

The β-escin cytotoxicity assessed on HaCaT cells by MTT assay showed a non-toxic effect at the highest concentration tested, a result quite promising compared to other plant extracts and also confirmed by in vivo experiments in *G. mellonella*. *G. mellonella* is an ideal infection model for in vivo antifungal drug screening and evaluation because it is easy to use, susceptible to infection, and widely proposed nowadays to avoid the ethical problems associated with mammalian models while also reducing the cost of experiments.

In this study, the survival rate of infected larvae in the drug combination group was significantly improved compared to the control group and drug monotherapy groups. The results showed that drug combination treatment was more effective than drug monotherapy for *C. glabrata* growth inhibition. Therefore, the in vivo data further confirmed the in vitro antifungal effect of this drug combination and reported the potential efficacy of this drug combination against resistant *C. glabrata* in vivo. This study led to the conclusion that these data suggest that β-escin may be a good candidate for the development of new antimicrobial agents to treat fungal-resistant infections.

## 4. Materials and Methods

### 4.1. Candida Strains, Cell Lines, and Culture 

Two clinical isolates of multi-drug-resistant *C. glabrata* (named C18 and C27) previously identified on the basis of morphology and molecular identification in our previous study [32] and belonging to our collection of fungal strains, and a reference strain *C. glabrata* DSM11226 were used in this work. All strains were stored at −80 °C and subcultured on Tryptic Soy Agar (TSA) and cultured in Tryptic Soy Broth (TSB) supplemented with 1% *w/v* glucose (VWR Chemicals, Radnor, Pennsylvania, United States of America) before each experiment. The human keratinocyte cell line HaCAT obtained from the ATCC (American Type Culture Collection, Manassas, VA, USA) was grown in Dulbecco’s Modified Eagle Medium (DMEM, Sigma Aldrich), supplemented with 10% Fetal Bovine Serum, 1% L-glutamine, and 1% penicillin/streptomycin (Sigma Aldrich), in a humidified incubator at 37 °C and 5% CO_2_. 

### 4.2. Antifungal Agents and Preparation of Drug Stock Solutions

Itraconazole (ITZ), Ketoconazole (KET), and Fluconazole (FLC) were purchased from Sigma Aldrich (Saint Louis, MO, USA). Stock solutions were prepared at a final concentration of 500 μg mL^−1^ in dimethyl sulfoxide (DMSO) 5% *v/v*. A stock solution of β-escin, 95% purity (Phytolab Vestenbergsgreuth, Germany), was prepared at a final concentration of 200 μg mL^−1^ in DMSO 5% *v/v*.

### 4.3. β-Escin Cytotoxicity

β-escin cytotoxicity was evaluated by 3-[4,5-dimethylthiazol-2-yl]-3,5 diphenyl tetrazolium bromide (MTT) assay. HaCAT cells were seeded in a 96-well plate at a density of 2 × 10^4^ cell/well and incubated for 24 h. The day after, cells were treated with several β-escin concentrations ranging from 1 to 20 μg mL^−1^. After 24 h, MTT solution was added to each well, and plates were incubated for 4 h in a humidified incubator at 37 °C and 5% CO_2_. Then, the medium was removed and replaced with DMSO to dissolve the formazan crystals, and the absorbance was measured at 570 nm with a microplate reader (SYNERGYH4, BioTek, Inc., Winooski, VT, USA). The percentage of cell viability was reported and compared to the viability of untreated cells.

### 4.4. Disk Diffusion Test 

Susceptibility testing to azoles was conducted according to the Clinical and Laboratory Standards Institute guidelines (CLSI, document M44-A2, 2009) [33] by the disc diffusion method. All *Candida* strains were adjusted to 10^7^ cells mL^−1^ (OD_590_nm = 0.7) and swabbed onto the surface of the medium. Absorbent discs impregnated with 10 μL of FLC (25 μg), ITR (10 μg), and KET (10 μg) and placed onto the surface of inoculated plates. The interpretation of antifungals was done according to the guidelines, considering a zone diameter ≥ 19 mm sensitive, = 15–18 mm diameter intermediate, and ≤ 14 mm diameter resistant.

### 4.5. Determination of the Minimum Inhibitory Concentration (MIC) and Minimum Fungicidal Concentration (MFC)

The minimal inhibitory concentration (MIC) of β-escin was determined using the Clinical and Laboratory Standards Institute (CISI, M27-A3, and M60) standard micro-broth dilution method [34,35]. The MIC was defined as the minimum concentration of the compound able to inhibit 90% growth of *Candida* cells compared to the growth control. To determine the minimum fungicidal concentration (MFC), an aliquot of 5 μL from each optically clear well was transferred onto TSA plates and incubated at 37 °C for 24 h. The MFC was the smallest concentration of the compound that inhibited growth on the agar plates. The MFC/MIC ratio was calculated to determine whether the substance had fungistatic (MFC/MIC ≥ 4) or fungicidal (MFC/MIC < 4) activity. All tests were performed in triplicate. 

### 4.6. Time–Growth and Time–Kill Curve Assays 

To construct the respective time-growth curves, the two *C. glabrata* clinical strains (C18 and C27) as well as the DSM11226 strain at an initial concentration of 1·× 10^6^ CFU·mL^−1^ were inoculated in the TSB-glucose medium containing β-escin at concentrations equivalent to 1/2 × MIC, 1 × MIC, and 2 × MIC and incubated at 37 °C for 24 h. Growth was determined by absorbance at 590 nm and monitored for 24 h. Curves were plotted to record the growth of each strain at each time point and compared to negative control curves. The fungicidal/fungistatic properties of β-escin were assessed using a time-kill study adapted from a protocol described earlier [32]. Briefly, for each tested organism, aliquots of 1 mL collected from the yeast cultures at intervals of 0, 8, 12, 16, and 24 h, were properly diluted and pipetted onto Petri dishes containing TSA-glucose. Plates were incubated at 37 °C for 24 h to determine the colony-forming units (CFU) and the viable cells in the sample. The experiment was carried out in triplicate for each strain.

### 4.7. Evaluation of the Activity of β-Escin against Candida Biofilms 

Inhibition of biofilm formation was determined in 96-well flat-bottomed plates as reported previously [32], adding to each 100 μL of fungal suspension in RPMI-1640 (10^6^ cells mL^−1^) and 100 μL of β-escin at sub-MIC concentrations of 0.5, 1, 5, 10, and 20 μg mL^−1^. The cultures were incubated at 37 °C for 24 h. Then, non-adherent cells were removed, and the wells were washed three times with PBS. Total biofilm mass was quantified using the crystal violet (CV) staining method. Briefly, biofilm was fixed at 37 ^◦^C for 1 h and then 200 μL of CV (0.2% *v/v*) was added to the well for 15 min. Biofilm was resuspended by adding 300 μL of 30% (*v/v*) acetic acid. The absorbance was quantified at 570 nm using a microtiter plate reader, as described previously [36]. The percent inhibition was calculated by normalizing each value with respect to the untreated control.

To assess the eradication capability of β-escin on a mature pre-formed biofilm, the compound at the same concentrations reported above was added, and 96-well plates were incubated for another 24 h at 37 °C. The biofilm eradication ability of β-escin was evaluated by the crystal violet colorimetric assay.

### 4.8. qRT-PCR Analysis 

Cells of biofilm grown together with β-escin (5 μg mL^−1^) at 37 °C for 24 h were scraped and washed in PBS as previously reported [37]. Total RNA was isolated using the Direct-zolTM RNA Miniprep Plus Kit (ZYMO RESEARCH, Irvine, CA, USA) according to the manufacturer’s instructions, and cDNA was obtained by reverse transcriptase (Bio-Rad, Milan, Italy) reaction using 1 μg of RNA. qRT-PCR was performed with 1 × SensiFAST TM SYBR Green master mix (total volume of 10 μL) (Meridiana Bioline) in an AriaMx Real-Time PCR instrument (Agilent Technologies, Inc., Milan, Italy) according to the manufacturer’s instructions. Fluorescence was measured using Agilent Aria 1.7 software (Agilent Technologies, Inc.). The expression of each gene was analyzed and normalized against the *ACT1* gene using REST software (Relative Expression Software Tool, Weihenstephan, Germany, version 1.9.12) based on the Pfaffl method [38,39]. The primer sequences used are listed in Table S1.

### 4.9. FACScan Analysis 

To analyze the membrane integrity of *C. glabrata* cells during the inhibition of biofilm by β-escin at the concentrations of 5 µg mL^−1^, 10 µg mL^−1^, and 20 µg mL^−1^, flow cytometry was used. After 18 h exposure, cells were harvested by centrifugation, washed three times with PBS, and 1 μg mL^−1^ of the final concentration of propidium iodide (PI) (Sigma Aldrich) was added at room temperature in the dark. After 30 min, the excess of PI was removed by washing cells with PBS, and flow cytometry analysis was performed using a FACScan (BD Accuri™ C6 Flow Cytometer Biosciences). Fungal cells incubated with PI without β-escin were the negative control.

### 4.10. Measurement of Reactive Oxygen Species Levels

Intracellular reactive oxygen species (iROS) were investigated using the fluorescent dye 2′,7′-dichlorofluorescein diacetate (DCFH-DA) (Molecular Probes, Eugene, OR, USA) and mitochondrial-specific ROS using MitoSOX Red (Molecular Probes, Eugene, OR, USA). After the inhibition of biofilm formation with 5 μg mL^−1^ of β-escin, *Candida* cells were collected, washed, and 10 mM H_2_DCFDA or 5 M MitoSOX Red were added for 1 h and 30 min, respectively, incubating cells at 37 °C in the dark. Fluorescence intensities (excitation and emission of 488 and 540 nm, respectively) were quantified by a microtiter plate reader (Synergy H4; BioTek Instruments, Inc, Colmar, France.).

### 4.11. Evaluation of the Synergistic Potential of β-Escin and Antifungal Drugs Associations against BIOFILMS 

In order to verify the combined effect of β-escin with FLC, ITZ, and KET on *Candida* biofilms, checkerboard microtiter assays were performed, as described previously [40,41]. The fractional FICI was calculated for each agent by dividing the inhibition concentration of the antifungal combination by its minimal biofilm inhibiting concentration (MBIC) value. The calculation formula for the FICI model is as follows:

FICI = MIBC_A\ combined_/MIBC_A alone_ + MBIC_B combined_/MBIC_B alone_

FICI ≤ 0.5 represents synergy of two drugs. No interaction occurred at 0.5 < FICI < 4.0, and antagonism occurred at FICI ≤ 4.0 [42]. Experiments were performed in triplicate.

### 4.12. Determination of In-Vivo Antifungal Effects by G. mellonella Infection Model 

The pathogenicity of *C. glabrata*, the toxicity, and the anticandidal effect of β-escin, azoles, and their combination were assessed using the *Galleria mellonella* killing assay as previously described. The 20 *G. mellonella* larvae in each group, with a body weight between 200 and 300 mg, were injected directly into the hemocoel right proleg region using a 50 µL Hamilton syringe with a 26 *g* needle. The experiments were performed in triplicate and only on the C18 strain.

Larval killing assays were carried out at 37 °C using different inocula of 10^5^, 10^6^, and 10^7^ yeast cells/larva, as previously described [43] and death was monitored daily over 4 days by visual inspection of the color and lack of movement after stimulation. 

Toxicity assays were performed by injecting 0.5 or 1.5μg mL^−1^ of β-escin 5 μg mL^−1^, FLC, 5 μg mL^−1^ KET, and 10 μg mL^−1^ ITZ. Toxicity was also tested on the combination of β-escin/azoles based on the FICI results of Esc/FLC, Esc/ITZ, and Esc/KET.

To evaluate the in vivo capacity of β-escin alone or in combination with azoles to avoid *C. glabrata* infection, larvae were inoculated with 10 μL of the selected concentration of yeast suspension via the last left proleg, and after 2 h of incubation, larvae were injected with each compound or combination. In another set of experiments, to check the ability of compounds to prevent infection, larvae were injected with each compound or combination and after 2 h with the yeast suspension. Groups of larvae untreated with the yeast or drugs served as a blank control group.

### 4.13. Statistical Analysis 

Statistical analysis was carried out using GraphPad Prism Software (version 9.00 for Windows, GraphPad Software, La Jolla, CA, USA, www.graphpad.com, accessed on 20 June 2023). The results were shown as the mean values ± standard deviation. 

For molecular analyses, relative expression ratios greater than ± 1 were considered significant (*p-*Value < 0.05 *t*-test). One-way ANOVA followed by Tukey’s test were used for the measurement of reactive oxygen species levels and for the checkerboard assay. Survival curves were plotted using the Kaplan–Meier method and the log-rank (Mantel—Coxtest) test.

## 5. Conclusions

The formation of *C. glabrata* biofilm cells enhances their capacity to evade the host defense and to resist conventional antimycotics. The current study explained that β-escin at sub-MIC concentration is able to inhibit and eradicate biofilms, inducing ROS activation and reducing various virulence factors. Gene expression analysis revealed that β-escin downregulated *ERG11, ALS3,* and *CDR1,* reducing the probability of developing antifungal resistance. The efficacy of β-escin targeting biofilm-associated virulence is improved by the synergistic strategy in vitro and in vivo. In conclusion, β-escin offers great potential to reduce azole resistance in fungi under in vitro and in vivo conditions, suggesting that its use in combination with common azoles for *Candida* infection is a potential therapy, even if further investigations are needed to clarify its potential clinical application.

## Figures and Tables

**Table 1:** Diameter of inhibition zone according to CLSI, document M44-A2, 2009 and antimycotic resistance profiles (S: susceptible; I: intermediate, R: resistant) of the reference DSM 1226 strain and clinical isolates C18 and C27 used in the study.

	ITZ	KET	FLC
	10 µg	10 µg	25 µg
	(Ø mm)		
DSM	25 (S)	25 (S)	20 (S)
C18	10 (R)	9 (R)	7 (R)
C27	22 (I)	16 (I)	15 (R)

**Table 2:** Antifungal activity of β-escin against *C. glabrata* DSM 1226 strain and clinical isolates (C18 and C27), expressed as minimal inhibitory concentration (MIC) and minimum fungicidal concentration (MFC). The ratio MFC/MIC < 4 indicates a fungicidal effect.

	MIC	MFC	MFC/MIC	
	(µg mL^−1^)		Ratio	
DSM	80	160	2.0	fungicidal
C18	40	100	2.5	fungicidal
C27	50	100	2.0	fungicidal

## Data Availability

Not applicable.

## Supplementary Materials

The following supporting information can be downloaded at: https://www.mdpi.com/article/10.3390/antibiotics12071210/s1, Table S1: Gene-specific primers used for real-time RT-PCR [44,45,46,47].
