# Supplementary material for: Effect of Escin Alone or in Combination with Antifungal Agents on Resistant Candida glabrata Biofilms: Mechanisms of Action
Source: Antibiotics (Basel). 2023 Jul 20;12(7):1210. doi: 10.3390/antibiotics12071210 (PMC10376425; doi:10.3390/antibiotics12071210)
Supplement: Supplementary file 1 [file antibiotics-12-01210-s001.zip › antibiotics-2500356-supplementary.pdf]

Table S1. Gene-specific primers used for real-time RT-PCR.

Gene name	Acronym	Primer name	Sequence (5'→3)	TM (°C)	Amplicon Length (bp)	Reference
Actin	*actin*	C.glabrata_actin_F<br>C.glabrata_actin_R	TTGCCACACGCTATTTTGAG<br>ACCATCTGGCAATTCGTAGG	57.01<br>57.67	225 bp	[1]
Ergosterol biosynthesis enzyme	*ERG11*	C.glabrata_ERG11_F<br>C.glabrata_ERG11_R	ATTGGTGTCTTGATGGGTGGTC<br>TCTTCTTGGACATCTGGTCTTTCA	60.56<br>59.65	92 bp	[2]
Agglutinin like-sequence 3	*ALS3*	C.glabrata_ALS3_F<br>C.glabrata_ALS3_R	CTGGACCACCAGGAAACACT<br>GGTGGAGCGGTGACAGTAGT	59.53<br>61.53	226 bp	[3]
Genes encoding efflux pumps	*CDR1*	C.glabrata_CDR1_F<br>C.glabrata_CDR1_R	TAGCACATCAACTACACGAACGT<br>AGAGTGAACATTAAGGATGCCATG	60.30<br>59.11	148 bp	[2]
High-osmolarity glycerol1	*HOG1*	C.glabrata_HOG1_F<br>C.glabrata_HOG1_R	GACTTGTGGTCTGTGGGTTG<br>ACATCAGCAGGAGGTGAGC	58.69<br>59.40	125 bp	[4]

References

[1] A. Cortegiani, G. Misseri, T. Fasciana *et al.*, "Epidemiology, clinical characteristics, resistance, and treatment of infections by Candida auris," *J Intensive Care,* vol. 6, pp. 69, 2018.

[2] S. G. Whaley, E. L. Berkow, J. M. Rybak *et al.*, "Azole antifungal resistance in Candida albicans and emerging non-albicans Candida species," *Frontiers in microbiology,* vol. 7, pp. 2173, 2017.

[3] M. C. Leite de Andrade, M. A. Soares de Oliveira, F. d. A. G. d. Santos *et al.*, "A new approach by optical coherence tomography for elucidating biofilm formation by emergent Candida species," *PLoS One,* vol. 12, no. 11, pp. e0188020, 2017.

[4] R. Rajendran, L. Sherry, C. J. Nile *et al.*, "Biofilm formation is a risk factor for mortality in patients with Candida albicans bloodstream infection—Scotland, 2012–2013," *Clinical Microbiology and Infection,* vol. 22, no. 1, pp. 87-93, 2016.
